# Parallel Chords: an audio-visual analytics design for parallel coordinates

**DOI:** 10.1007/s00779-024-01795-8

**Published:** 2024-05-03

**Authors:** Elias Elmquist, Kajetan Enge, Alexander Rind, Carlo Navarra, Robert Höldrich, Michael Iber, Alexander Bock, Anders Ynnerman, Wolfgang Aigner, Niklas Rönnberg

**Affiliations:** 1 Linköping University, Linköping, Sweden; 2 St. Pölten University of Applied Sciences, St. Pölten, Austria; 3 University of Music and Performing Arts Graz, Graz, Austria

**Keywords:** Audio-visual analytics, Sonification, Parallel coordinates, User evaluation

## Abstract

One of the commonly used visualization techniques for multivariate data is the parallel coordinates plot. It provides users with a visual overview of multivariate data and the possibility to interactively explore it. While pattern recognition is a strength of the human visual system, it is also a strength of the auditory system. Inspired by the integration of the visual and auditory perception in everyday life, we introduce an audio-visual analytics design named *Parallel Chords* combining both visual and auditory displays. *Parallel Chords* lets users explore multivariate data using both visualization and sonification through the interaction with the axes of a parallel coordinates plot. To illustrate the potential of the design, we present (1) prototypical data patterns where the sonification helps with the identification of correlations, clusters, and outliers, (2) a usage scenario showing the sonification of data from non-adjacent axes, and (3) a controlled experiment on the sensitivity thresholds of participants when distinguishing the strength of correlations. During this controlled experiment, 35 participants used three different display types, the visualization, the sonification, and the combination of these, to identify the strongest out of three correlations. The results show that all three display types enabled the participants to identify the strongest correlation — with visualization resulting in the best sensitivity. The sonification resulted in sensitivities that were independent from the type of displayed correlation, and the combination resulted in increased enjoyability during usage.

**Supplementary Information:**

The online version contains supplementary material available at 10.1007/s00779-024-01795-8.

## Introduction

In the context of analyzing multivariate data, visual analytics has proven to be effective for the detection of patterns and trends between variables. Parallel coordinates [44, 88] is a widely used visualization technique for analyzing multivariate data, where individual variables are presented as vertical axes that are evenly spaced parallel to each other. Data items are represented by polylines that intersect the axes at the value of the respective data item.

Even though the parallel coordinates technique is well-suited to visualize multivariate data, a number of challenges exist [37]. The individual axes of parallel coordinates being positioned next to each other allow one variable to be compared to at most two direct neighbors at a time. Axis order algorithms can be applied to set the optimal axis order to reveal a certain pattern, e.g., clusters [7], but the problem still persists when making more bivariate comparisons than the parallel coordinates plot allows. To overcome this limitation, parallel coordinates and other multivariate visualizations are often part of a multiple-view system to provide additional perspectives of the data. One approach is to display several parallel coordinates plots simultaneously as a matrix, showing all pairwise relations for the variables of the dataset [38]. Another approach is to accompany the parallel coordinates plot with bivariate visualizations. This allows displaying more variable comparisons within the same context, and can also make use of the strengths of different types of visualizations. For example, Li et al. [54] conducted a comparative user experiment for scatter plots and parallel coordinates and found that users can distinguish twice as many correlation levels in a scatter plot than in a parallel coordinates plot, and that users overestimate negative correlations with parallel coordinates. On the other hand, Kanjanabose et al. [47] showed that participants performed better with clustering and outlier detection tasks when using a parallel coordinates plot compared to using a scatter plot. This shows that different types of visualizations can complement each other when used in conjunction. However, presenting several types of visualizations simultaneously in a multiple-view system can lead to a loss of context and focus when switching between the views [59]. It also leaves less space for each visualization on the display, which especially impacts displays with limited screen size. Therefore, other ways of conveying additional bivariate information for multivariate visualizations should be explored. The added information and perspectives could instead be conveyed by another modality, such as the auditory, which has specific differences from the visual modality. Numerous studies on sonification as a technique for auditory representation of data have demonstrated its potential for the recognition of coherent patterns [21, 31, 60].

In this article, we investigate how the addition of the auditory modality through sonification can be beneficially used in conjunction with a parallel coordinates visualization by creating an audio-visual analytics design, named *Parallel Chords*. This is demonstrated by presenting how the sonification would convey different prototypical patterns, while also demonstrating it in a usage scenario. As a first step to validate the approach, a controlled experiment was conducted towards the most foundational type of patterns, i.e., correlations (positive and negative), to evaluate the sensitivity thresholds and experience of participants when distinguishing similar correlations through visualization, sonification, and by using both in combination. Through the demonstration of an audio-visual analytics design and analysis of experiment results, the following contributions are presented:

- An audio-visual analytics design for parallel coordinates, which is demonstrated with the use of prototypical data patterns and a usage scenario.
- Quantitative and qualitative evaluation results of participants distinguishing similar correlations using parallel coordinates, an auditory scatter plot, and the combined usage of both.

The results of this study will support both the visualization and the sonification communities to better understand the implications of audio-visual designs, what to consider during sound design, and in what context to combine sonification and visualization.

## Related work

Previous research from both the visualization and the sonification areas is relevant to this work. We present an overview of visualization idioms for multivariate data, and specifically what solutions exist to mitigate the challenges of the parallel coordinates technique. We present how sonification can complement visualization, and what audio-visual analytics designs currently exist.

### Visualization of multivariate data

Visualization idioms for multivariate data focus on datasets that encompass a large number of items, three or more quantitative variables, and no further relational information between the items. Numerous visualization idioms have been designed for such data [80, 87] and we will compare parallel coordinates plots to six idiom families. *Axis-based* idioms represent data items in a layout that maps variables to axes. With points, the scatter plots for each pair of variables are shown together in a grid, the scatter plot matrix, or sequentially one after another, the grand tour [4]. Both arrangements of the scatter plots depict each data item as multiple point marks either spatially or temporally and require the user to mentally connect the marks for multivariate patterns. In the parallel coordinates plot [44, 88], each data item is represented as one polyline, which, at least in principle, visually connects all the variable values. A radar plot is comparable to the parallel coordinates plot but places the axes in a radial arrangement. Polyline-based idioms allow the analysis of bivariate patterns best between neighboring axes. All axis-based idioms preserve the quantitative distribution of the variable values but they can suffer from overplotting. *Table-based* idioms such as Table Lens [66] simply follow the visual layout of a spreadsheet’s rows and columns while densely encoding data values, e.g., as a bar chart. With interactive sorting, the user can identify patterns between two or more variables. They avoid overplotting but the y-position does not directly express a variable value. A third family of idioms algorithmically transforms multivariate data to simple visual marks. Dimension reduction algorithms like multi-dimensional scaling, primary component analysis, or stochastic neighbor embedding can reduce the dataset to the two dimensions of a scatter plot [76]. The RadViz [42] or Dust & Magnet [93] idiom applies a force-based metaphor so that point marks for items are positioned closer to the variable marks, for which they have a large value. The Andrews’ curves [2] idiom displays a multivariate item as the plot of a finite Fourier series. *Glyph-based* idioms such as profile glyphs, stick figures, or Chernoff faces represent each data item with a visual entity that encodes multiple variables to the visual channels of one or more visual marks [8, 34]. Transformation-based and glyph-based idioms focus on the holistic representation of the data items whereas parallel coordinates also display patterns across one or more variables. *Pixel-based* idioms such as VisDB [49] and *nested* visualizations such as mosaic plots and dimensional stacking [52] rely on some relational information such as a temporal dimension or categories to arrange the multivariate data and are thus not directly comparable to parallel coordinates.

### Enhancements of parallel coordinates

While parallel coordinates plots provide an overview of multivariate data expressing all variable values along scaled axes, they have limitations from overplotting and can play their strengths best for bivariate patterns between two variables that are displayed on neighboring axes. These limitations can be addressed, to some extent, by interactively filtering items and changing the order and direction of axes. The effects of overplotting can be mitigated by histograms on top of axes, semi-transparent polylines, rasterization that preserves line orientations [58], or showing clusters of items as bands [33, 61]. Various algorithmic approaches, e.g., [3, 20, 64, 92], propose an axes order that reduces overplotting or better reveals patterns such as clusters. Blumenschein et al. [7] identified 32 reordering approaches and found out that there is a trade-off between these goals. Furthermore, the users’ background knowledge about the data and the task may still call for a different combination of variables to be analyzed together.

Other visualization idioms derived from parallel coordinates abandon the sequential arrangement of the axes in order to better show bivariate patterns. TimeWheel [81], 3D TimeWheel [82], and CMRPC [46] place one axis in the center and connect it with all other axes that are positioned around it. In the 2D version, the axes cannot be placed parallel. Lind et al. [55] replicated these axes and arranged them as polygons around a central variable. These show all bivariate combinations of variables with a pair of parallel axis in 2D. The Parallel Scatterplot Matrix [85] can be interactively rotated from a scatter plot matrix into a multi-row parallel coordinates plot. The Parallel Coordinates Matrix [38] combines as many rows of parallel coordinates plots as needed to display all pairs of variables as neighbors. Claessen and van Wijk [15] give users the flexibility to create custom layouts by drawing and linking axes on a 2D canvas. All these derived approaches have drawbacks similar to the scatter plot matrix: the data item is no longer represented by a single visual mark and the subdivision of display space decreases the visual resolution. Some of the approaches are further restricted by rotated axes or a 3D projection.

### Sonification and audio-visual analytics

Sonification is the auditory equivalent to visualization, i.e., the transformation of data into sound or the mapping of data characteristics to auditory channels [39, 65]. Sonification can be used for data exploration and there are a number of studies that evaluate auditory graphs [30, 57, 60, 79]. It has also been demonstrated that sonification can support visual perception [1, 70, 71], and various auditory channels can be successfully linked and related to visual channels [17, 21, 27, 57, 86]. Sounds, in sonification, can convey a multitude of information to listeners quickly [83], without adding visual clutter [13]. This suggests that sonification and visualization can fruitfully be combined, and previous studies have explored this combination [11, 21, 23, 25, 30, 41, 48, 57, 60, 67, 69]. While these studies indicate that using sonification together with visualization supports a user in various data analysis tasks, also theoretical bridges between the visualization and the sonification communities exist. Theoretical constructs from visualization research — the “spatial substrate,” the “mark,” and the “channels” [59] — have been adopted to sonification. The theoretical framework characterizes sonification using time as its “substrate” and zero-dimensional and one-dimensional “auditory marks” that use “auditory channels” to control their auditory appearance [24, 26].

Auditory scatter plots, where data attributes are mapped to note onset, duration, and pitch, can provide information resulting in almost identical estimates of correlation magnitude and correlation coefficients as for visual representations [31]. Judgments of correlation magnitude are also similarly affected by single outliers in visual and auditory scatter plots [31]. Auditory scatter plots where density levels have been mapped to auditory channels have also been proven to successfully provide information about density levels as well as data distribution and clustering [68, 91]. Sonification can also be used to support the perception of visually dense areas in cluttered visual displays, both scatter plots and parallel coordinates representations, by sonifying density levels as well as different datasets in the visual representation [72]. Furthermore, sonification can be used together with parallel coordinates plots for perception-based classification of individual data records in a relational dataset [62, 63]. Finally, sonification has been proposed to be able to complement parallel coordinates in terms of visual cluster overlapping, visual representations in general for high dimensionality data, challenges for the visual perception in color distinction, and limitations by screen resolution [36]. Based on these findings we argue that sonification can be successfully used as a supplement to visual representations, and can be used to discover classes of data and data features (see further discussions in [30]).

**Fig. 1:**
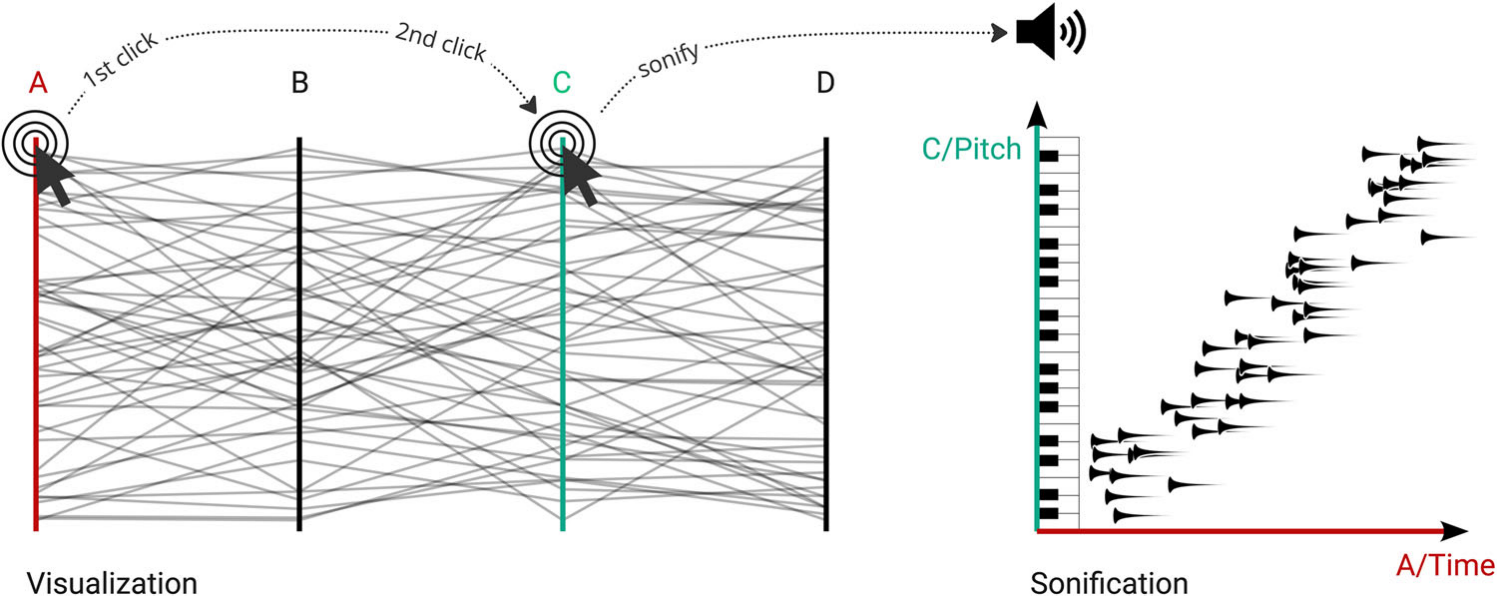
Illustration of the audio-visual analytics design *Parallel Chords* when exploring the relationship of non-adjacent axes in a parallel coordinates plot. A user selects one axis (1) which is mapped to the temporal onsets of individual sounds in the sonification, and a second axis (2) which is mapped to the pitch of the individual sounds. In this case, the sonification conveys a positive correlation between axes *A* and *C* to the user represented via increasing pitches as time progresses

## Parallel chords

To explore the combination of sonification and visualization in the context of parallel coordinates, workshops were conducted to decide on the final design, which are described in Section 3.1. Based on these discussions, we created an audio-visual analytics design named *Parallel Chords*. It enables users to explore multivariate data through both visualization and sonification by interacting with the axes of a parallel coordinates plot. Through the *Parallel Chords* interface, the user can select two axes by sequentially clicking on two axis labels (see Fig. 1). The variable selected first is mapped to the temporal onset of auditory marks in the sonification, and the variable of the second selected axis is mapped to their pitch. As soon as the user has selected the second axis, the interrelationship between the two axes is sonified. After this, two new axes can be selected for comparison.

This section first summarizes our design process and then presents the visualization and sonification components of *Parallel Chords* in detail using the constructs of the unified theoretical framework for audio-visual analytics designs by Enge et al.  [24, 26]: *Visual marks* are placed in space (the *substrate* of visualization) using *channels* to encode information. *Auditory marks* (i.e., the individual sounds) are placed in time (the *substrate* of sonification) using *auditory channels* to encode information. In *Parallel Chords* each data item is represented both as a visual polyline and as an auditory mark that uses two auditory channels to encode information: the auditory mark’s onset time and its pitch. In Section 4, we demonstrate the design by applying it to commonly occurring patterns in visual analytics, and in Section 4.1, we present a usage scenario demonstrating how the design can be used to convey information about non-adjacent axes in a parallel coordinates plot.

### Design process

To investigate how sonification could support a parallel coordinates visualization, workshops were conducted where two visualization researchers and three sonification researchers, which are co-authors of this article, were interviewed as a group around how sonification could be used to benefit the visualization. The first workshop was an ideation workshop to identify challenges and generate ideas related to the integration of sonification with parallel coordinates. The second workshop, again within our group, was a concept workshop discussing different prototypical implementations that tackle the challenges of parallel coordinates identified in the ideation workshop. Both these workshops were led by the two first authors of this article and were conducted as semi-structured group interviews. Additionally, we conducted individual workshops with one interaction design researcher and one visualization researcher with a specific interest in parallel coordinates. These meetings were conducted as semi-structured interviews as well and provided an external and well-informed perspective onto our design ideas.

The notes that were taken during all workshops were categorized by the two first authors to summarize the outcome. The main take-aways from the ideation workshop was that sonification had the possibility to prevent specific shortcomings of parallel coordinates. One of the identified shortcomings concerned visual clutter due to the overplotting of an abundance of data items. Another challenge was the axis ordering problem and the limitation of pair-wise comparison of axes, which led to non-adjacent axes not being comparable. A number of sonification mapping strategies were created to concretize the ideas from the ideation workshop, which would aid in the shortcomings of the visualization. These mapping strategies were realized as sonification concepts and were demonstrated at the concept workshop.

The mapping strategies focused on the two main challenges of parallel coordinates that were highlighted in the ideation workshop, namely overplotting and axis ordering. The mapping strategies for mitigating axis ordering focused on representing several data dimensions at the same time or in quick succession to get an overview of the dataset through the sonification. By mapping each attribute in a dataset to an individual auditory channel, it would enable a user to become aware of patterns in the dataset by listening to changes in the sonification. Another mapping approach was to use the same sonification mapping for each attribute but spatialize them to the corresponding axis in the parallel coordinates plot to be able to distinguish between them.

The mapping strategies for overplotting focused on representing the lines in the parallel coordinates plot in an auditory manner to aid in detecting patterns in a large dataset. One suggested mapping strategy was to convey the angle of the lines in a parallel coordinates plot through the sonification to perceive correlations in the dataset. Another mapping strategy involved a dynamic line selection, where lines in the parallel coordinates plot would only be sonified if they diverged from the general pattern between two axes.

While many of the mapping strategies had the potential of complementing a parallel coordinates visualization, we decided to use an established sonification mapping approach to act as an initial exploration of the combined design. Based on the discussions during the workshops, we decided for combining and studying mappings that are well researched individually, also due to the fact that we are investigating a relatively new field with this study. Therefore, the sonification was created to work as an auditory equivalent of a scatter plot, which is an established and well researched technique in the sonification community [30, 31, 57, 60, 79].

### Visualization design

The visualization design consists of a standard parallel coordinates plot which was created by using the Data-Driven Documents (D3.js) information visualization library [9]. The parallel coordinates plot contains generic axis labels without tick marks to put focus on the data patterns themselves rather than specific data values. The lines are colored in black and were drawn with 30% opacity to allow blending of the lines. Users explore different variable relations by dragging the axes of the parallel coordinates plot. As this design will be used in a controlled experiment that investigates the identification of global data patterns such as correlations or outliers, it does not provide interaction for filtering or details on demand. This includes brushing and zooming of axes that filter the data range of an axis and highlighting data lines that give additional details of each data item. However, these common types of interactions should be provided when applying the design in a real-world use case and evaluating it in future application-oriented studies.

### Sonification design

While the parallel coordinates plot displays all attributes at once in a certain axis order, the sonification works as an auditory scatter plot of two user-selected attributes. Onset time is used to convey the values of the first selected axis, where lower values lead to earlier onset times for the respective auditory mark. This is scaled in relation to the value range of the variable, such that value gaps in the data are noticeable as pauses. The overall playback duration of the sonification is set to range between one and two seconds. The auditory channel pitch is used to represent the values of the second selected axis, where a higher data value results in a higher pitch for the auditory mark, creating a positive polarity mapping. The pitch ranges from MIDI note number 55 (G3, fundamental frequency 196 Hz) to 91 (G6, fundamental frequency 1567.98 Hz). Together with the onset mapping, the change of pitch over time enables the user to detect auditory patterns in the data. A positive correlation between two variables, for example, would result in a sequence of auditory marks with later marks having higher pitch than earlier ones. In the course of this article, the axis that is used to sort the onset times will be called the *time axis*, and the axis that is used to sort the pitches of the individual auditory marks will be called the *pitch axis*.

The synthetic model of a mallet instrument comparable to a marimbaphone is used for the sonic representation of the auditory marks. The sound design was chosen for its clear distinction of temporal onsets, short decay time, and aesthetic qualities. The clear temporal onset of a mallet instrument aids in discerning individual data points when played in rapid succession. The short decay times of the individual sounds help to avoid temporal clutter during the sonification. If the chosen instrument needed a longer decay time to sound plausible, the individual sounds would soon mask each other. Using a sound design that resembles a real instrument was a dedicated decision. The timbre of a real instrument could be perceived as more aesthetic and therefore could increase its acceptability in comparison to the characteristics of a pure sine wave. Choosing such a sound design is also in line with all four design criteria for effective sonification design recently presented by Groß-Vogt et al. [35]. The four criteria are (1) to use easily perceptible sounds, (2) not to contradict data metaphors, (3) to follow a natural mapping, and (4) to use sounds appropriate to the task. The sonification model was implemented using SuperCollider [56], a real-time sound synthesis software environment that is commonly used for sonification purposes [12].

## Prototypical patterns

**Fig. 2:**
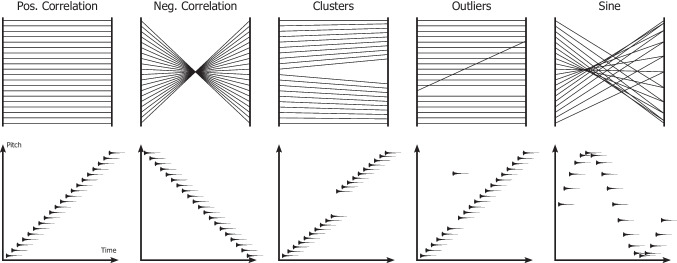
Prototypical patterns: positive and negative correlations, clusters, outliers, and, additionally, a sine function. The patterns are displayed in a parallel coordinates plot and in an illustrated auditory scatter plot to demonstrate the audio-visual mapping of *Parallel Chords*. While the left axes of the individual parallel coordinates plots are used as the *time axes* of the auditory scatter plots, the right axes of the parallel coordinates plots are used as the *pitch axis*. A positive correlation, therefore, results in a sound sequence of individual tones rising in pitch over time. See *Video 1* to listen to the sonification of the patterns, including more variations and noisy versions

To demonstrate *Parallel Chords*, we present commonly occurring types of data patterns [37] to serve as examples for the design. These patterns are positive and negative correlations, clusters, outliers, and sine. A compilation of the prototypical patterns in a parallel coordinates plot and in an illustrated auditory scatter plot can be seen in Fig. 2. The parallel coordinates plot and scatter plot create distinctly different outputs for each type of pattern. Since the sonification adopts an auditory scatter plot approach, different perspectives of the data can be gained by using both the visualization and the sonification simultaneously. An audio-visual demonstration of the patterns shown in Fig. 2 and additional examples can be viewed in *Video 1*.^1^ The video includes more variations for each type of pattern compared to what is shown in Fig. 2, and also includes versions of the patterns where noise has been added to convey how the sonification behaves for less clear patterns. The video displays all of the patterns sequentially and uses an arrow to guide the viewer during the playback of the sonification. The rest of this section describes each type of pattern with respect to its statistical meaning, what variations of the pattern exist, how it could be identified with the audio-visual design, and how a user could benefit from using *Parallel Chords* to detect the patterns.

### Correlation

Correlation conveys to which degree two variables are linearly related to each other, ranging from perfect negative to perfect positive correlations. Positive and negative correlations are polar opposites from a mathematical perspective and create distinctly different types of patterns in a parallel coordinates plot. A perfect positive correlation is visually displayed as parallel lines between two axes, while a negative correlation is displayed in the shape of an “X” or diabolo [54]. Since the sonification has an auditory scatter plot approach, the two types of correlations are more alike, where a positive correlation is identified as a sequential increase of pitch of the auditory marks, while a negative correlation is identified as a sequential decrease in pitch. To determine the strength of a correlation, positive or negative, a user would analyze how sorted the auditory sequence is regarding pitch. The sonification aids in estimating correlations by offering a second perspective. The exploration of positive and negative correlations is what was studied in the controlled experiment of this study, presented in Section 5.

### Clusters

Clusters are groups of data items that share similarities with regard to at least one of their variables. In a parallel coordinates plot, a cluster is visually represented as a structure of lines that appear spatially grouped together, and usually creates several distinct visual patterns when many clusters are present. Auditory clusters can either be identified through the temporal or pitch grouping in the sonification, depending on the characteristics of the cluster. If clusters are present on the *pitch axis*, the distinction of the clusters is identified by a sudden and bigger difference in pitch. If clusters are present on the *time axis*, they are identified by a pause that separates groups of auditory marks. If clusters are occurring on both axes, a combination of these two effects is perceived. Since the two mappings of the sonification could be easier to distinguish between, compared to the visualization, it could support the user in detecting and identifying clusters in the dataset.

### Outliers

An outlier is characterized as a data item that differs substantially from the rest of the dataset. This can be due to it being outside of the general range of values in the dataset, or that it differs from the pattern that is displayed when comparing two variables with each other. An outlier is identified with the sonification by listening for a deviation in the overarching pattern of the dataset. Similarly to clusters, if an outlier is present on the *pitch axis*, it is identified by a sudden and big difference in pitch. If an outlier is present on the *time axis*, there will be a temporal pause separating the outlier from the rest of the auditory marks. Since the auditory modality has a high sensitivity to both temporal changes and changes in pitch [94], the sonification supports the user in detecting outliers in the dataset.

### Sine

Some types of patterns could be easier to identify audibly due to their inherent development over time, such as sine functions. Although these types of patterns are not as commonly occurring in real-life multivariate data, it gives an example of how the temporal perspective of the sonification can contribute to the analysis of the data. When visually inspecting the pattern of a sine function through a parallel coordinates plot, one might not associate the image with a sine function. Through the sonification, however, there is a signature sound of a periodical increase and decrease of pitch over time. Furthermore, the number of periods can be identified by counting how many times the auditory pattern is repeated.

### Usage scenario

The presented prototypical patterns have demonstrated the use of *Parallel Chords* for commonly occurring patterns in a parallel coordinates plot with two axes. The following usage scenario illustrates how *Parallel Chords* could be used in practice by a fictional analyst, Dr. B., exploring botanical data. While this fictional user story can not validate our design, it serves to clarify our vision for such a tool. Dr. B. has musical experience and is a data expert that has prior experience with using sonification and visualization to analyze and explore her data. She wears headphones and uses a standard screen in her office to interact with *Parallel Chords*. The supplemental material provides a video of the interactions that are relevant in the usage scenario of *Parallel Chords*.^2^

At the beginning of her analysis, Dr. B checks if the system is up and running by clicking axis D and E. She chose those two axes as this is were she sees two distinct clusters that are not overlapping. Therefore, she expects to hear a cluster of lower tones in the beginning, then a pause and a cluster of higher notes afterward. The system seems to work and Dr. B continues her investigation. From her previous experience using *Parallel Chords*, she knows that sometimes patterns can be more audible than visible. She is interested in the relationship between axis A and B and listens to them. The sonification reveals the presence of three clusters. After Dr. B. has heard the three clusters, she takes a closer look and now also sees them. For more detailed analysis, she decides to use a scatter plot at a later point and continues with her analysis using *Parallel Chords.* Dr. B is expecting axis A and D to hold clusters but does not know how many exist. From her experience, she knows that the relationship between A and D is only relevant if the two axes hold three or more clusters with each other. To quickly check the number of clusters between the two non-adjacent axes, without changing the visual view onto her data, Dr. B. sonifies their relationship and hears three distinct clusters. She now wants to understand the relationship better and drags axis D next to axis A to visualize their connection. The visualization helps her to understand that the lowest cluster is strictly separated from the other two, while the two remaining clusters slightly overlap each other.

In our scenario, Dr. B. listened to two visually adjacent axes (A and B) because she suspected a pattern that was not clearly visible to her. The sonification led her to take another look directed toward clusters which made her see them. We expect such an interaction between visualization and sonification to be dependent on prior experience and gained intuition using a tool. To better understand the occurrence of such phenomena, follow-up studies will be necessary as they are out of the scope of this article. Dr. B. also used the sonification in a complementary manner with the visualization by sonifying non-adjacent axes (A and D). By using only the sonification, non-adjacent axes can be explored separately from the visualization to aid the user in making multiple bivariate comparisons for the same axis, something that is otherwise a shortcoming with parallel coordinates. This can also be useful if an axes order algorithm has been applied to the dataset since axes comparisons can be made by the user while keeping the axis ordering of the visualization untouched. The user can explore each combination of axes with the sonification, and choose to confirm or sharpen their impression using the visualization by dragging the axes together. The user can also alternate between assigning the two axes of a pair to become either the *time axis* or the *pitch axis* to get an additional auditory perspective.

## User evaluation

As a first step to validate *Parallel Chords*, we performed a controlled experiment for the most foundational type of pattern, namely correlations (positive and negative). The participants were asked to identify the strongest out of three correlations. This experiment task was chosen to understand the participant’s ability to correctly interpret the mapping of the sonification and to study the participants’ sensitivity in distinguishing small differences in the presented data.

Positive and negative correlations were tested separately to find potential differences between distinguishing the strength of the two types of correlations. Participants were tasked with identifying the strongest correlation using three display types: *Visualization*, *Sonification*, and by using both in *Combination*. *Visualization* would be used as a benchmark for regular use with a parallel coordinates plot. *Sonification* reflects how a user would use *Parallel Chords* for non-adjacent axes since there would not be any visual representation of the correlations. *Combination* would reflect how *Parallel Chords* is used for adjacent axes with combined visual and auditory representations of the correlations and would capture the sensitivity threshold of the participants when using both modalities at the same time.

### Method

**Fig. 3:**
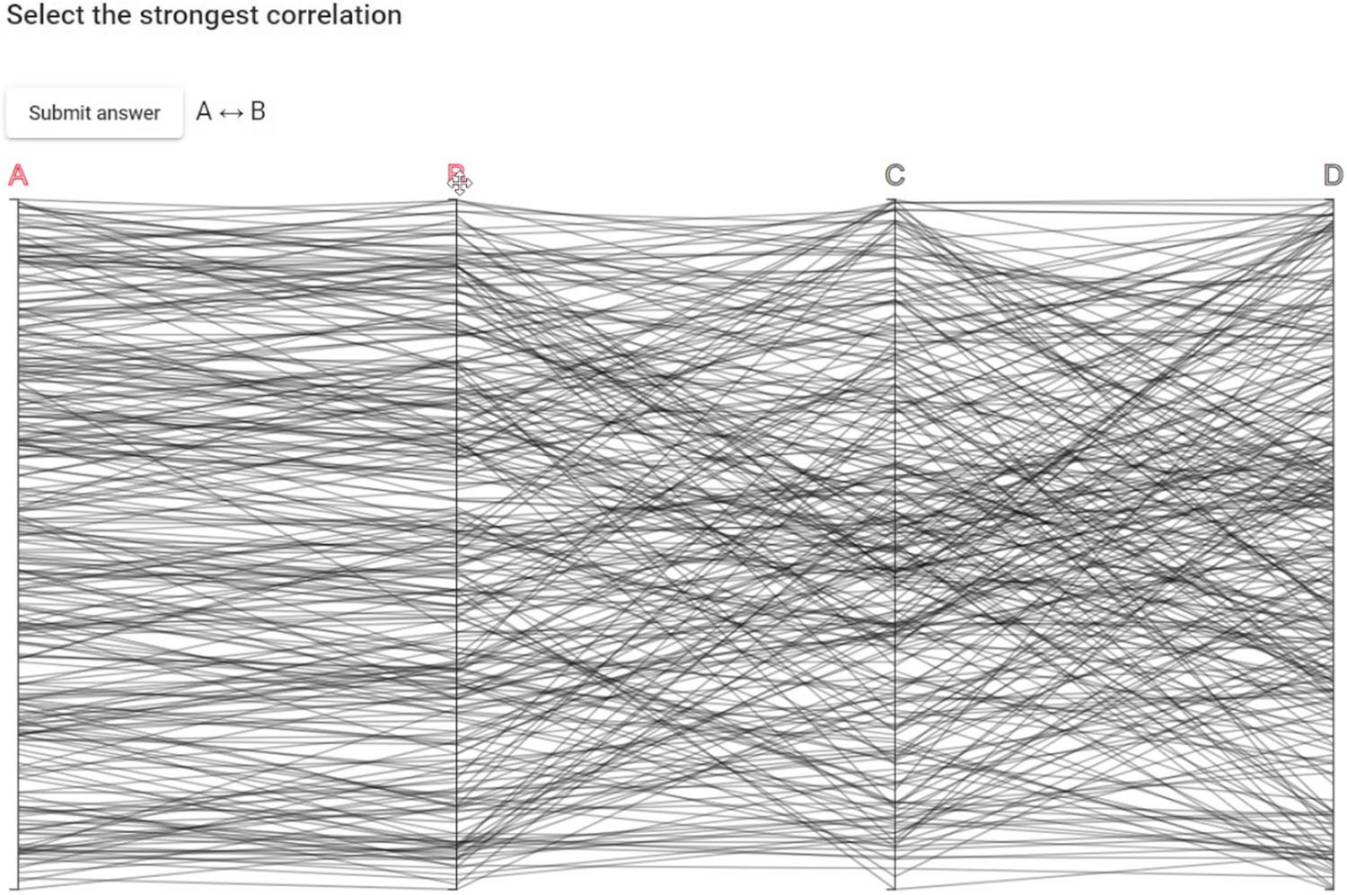
The experiment interface, where the participant has currently selected the correlation between axis *A* and *B* as their answer. The task is to select which out of the three axes *B*, *C*, or *D* holds the strongest correlation with axis *A*. Using *Sonification* the 300 polylines were not visible and the interface only consisted of the axes and their labels. See the tutorial video in the supplemental material for a video of the example

The experiment tasks were structured as “three alternative forced choice tests.” A parallel coordinates plot with four axes was presented, where the participant would select which out of the three axes *B*, *C*, or *D* had the strongest correlation with axis *A*. For *Visualization*, the participant would interact by dragging the axes, as is commonly done with parallel coordinates, to compare and select the strongest correlation. For *Sonification*, the polylines of the parallel coordinates plot were not visible and the participant clicked on either axis *B*, *C*, or *D* to listen to their correlations with axis *A*. The *time axis* for the sonification was always assigned to axis *A*, which meant that the participant only needed to assign the *pitch axis* to any of the other axes. For *Combination*, the participant used the dragging interaction which would trigger the sonification when an axis was released next to axis A. It was also possible to click on the currently compared axis to listen to the sonification again. A screenshot of the experiment interface can be seen in Fig. 3.

The sensitivity threshold of the participant was measured using a staircase test design [22, 53], where the difficulty of the tasks changed depending on the prior responses of the participant. The difficulty, in this context, relates to the difference in the correlation coefficient between the correct axis, which had the strongest correlation with axis *A*, and the two incorrect axes, which had a weaker correlation with axis *A*. Smaller differences in the strength of correlation result in a more difficult detection task. The staircase procedure followed a one-up two-down procedure [53], meaning that the difficulty would increase after responding correctly two times in a row, and decrease after responding incorrectly once. Each increase in difficulty would span two levels of difficulty, while each decrease would span only one level of difficulty. Furthermore, the difficulty would increase by three levels after responding correctly four times in a row to enable the participant to converge to their individual sensitivity threshold with fewer number of tasks. The staircase started with a low level of difficulty, i.e., with large differences between the correct and wrong answers, so that it can be assumed most participants would be able to correctly respond to the first few examples before reaching their individual thresholds. One staircase procedure was used for each of the negative and positive correlation tasks to be able to analyze the results separately. The positive and negative staircases were interleaved such that every other task displayed negative correlations and the tasks in between displayed positive correlations. Additionally, the task completion times and the number of interactions needed to submit an answer were recorded. Qualitative aspects were obtained from a questionnaire that contained questions to be answered before and after the experiment, capturing the participant’s subjective experience when performing the experiment. Pilot tests were conducted in preparation for the experiment to determine suitable correlation strengths and the number of tasks for each participant.

### Datasets

Synthetic data was created to be used in the controlled experiment. Out of the three selectable axes (*B*, *C*, *D*), one axis would hold a stronger correlation with axis *A*. Selecting that axis was the correct answer, and selecting one of the other two axes, holding a weaker correlation with axis *A*, was an incorrect answer. Equations 1, 2, 3, and 4 describe the generation of the vectors $v1-v4$ that were displayed on axis *A-D*. Vector *v*1 was always displayed on axis *A* and vectors $v2-v4$ were randomly assigned to axis *B, C*, and *D*.

Axis *A*, displayed as the left-most axis during the experiment, held the vector *v*1, a linearly spaced vector holding 300 entries with values between 0 and 1, that had the reference noise of 7% (defined here as Gaussian noise with $\sigma = 0.07$) added to it. The axis that held the strongest correlation with axis *A* (with a Pearson correlation of about 0.95) had the same properties as axis *A*, but since it was individually generated it would not hold identical values. The two other axes were both individually generated with additional added noise ($\Delta \sigma$), so that they had the same properties but with a weaker correlation with axis *A*.

1 $$\begin{aligned} v_1(i)= & {} \mathcal {N}(\frac{i}{300},\,\sigma ^{2}) \end{aligned}$$

2 $$\begin{aligned} v_2(i)= & {} \mathcal {N}(\frac{i}{300},\,\sigma ^{2}) \end{aligned}$$

3 $$\begin{aligned} v_3(i)= & {} \mathcal {N}(\frac{i}{300},\,(\sigma +\Delta \sigma )^{2}) \end{aligned}$$

4 $$\begin{aligned} v_4(i)= & {} \mathcal {N}(\frac{i}{300},\,(\sigma +\Delta \sigma )^{2}) \end{aligned}$$

where

$$\begin{aligned} i&\quad = 0,1,2,...,299\\ \mathcal {N}(\mu ,\,\sigma ^{2})&\quad = \text {normal distribution} \\ \sigma&\quad = \text {reference noise}~(0.07)\\ \Delta \sigma&\quad = \text {additionally added noise}~(0.02 - 20). \end{aligned}$$

The level of $\Delta \sigma$ determined the difficulty of the tasks. A lower $\Delta \sigma$ would result in more similar stimuli, which increased the difficulty of the task. 20 different levels of $\Delta \sigma$ were used to serve as the difficulty levels of the staircase test, ranging from $\Delta \sigma = 20\%$ (least difficult) down to $\Delta \sigma = 0.2$% (most difficult). Negatively correlated datasets were generated in the same manner, only that the selectable axes had their data entries inverted. Whenever a generated value happened to be out of range [0–1] due to added noise, a new random value with $\mu = \frac{i}{300}$ was generated and replaced with the original one. This was necessary for the datasets not to become sparsely populated around the edges of the axes after normalizing them to the same value range in the final step. The Jupyter Notebook we used to generate the datasets is available in the supplemental materials.

**Fig. 4:**
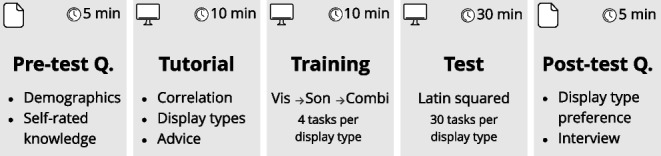
The five parts that were included in the experiment procedure, including how long each part took to complete. The questionnaires were done on paper. The tutorial, training, and test were done on a computer

### Procedure

The experiment procedure took participants around one hour to complete and was divided into five parts: pre-test questionnaire, tutorial, training, test, and post-test questionnaire (see Fig. 4). The experiment started with the participant filling in pre-test questions in a paper-based questionnaire concerning their age, gender, and possible perceptual impairments. Self-rated knowledge of the concept of correlation, their familiarity with parallel coordinates, their musical experience, and their familiarity with sonification were collected through a 5-point Likert scale. The experiment questionnaire can be viewed in the supplemental materials. The experiment proceeded on the computer with a tutorial on how to analyze correlations with visualization and sonification, respectively, and on how to interact with the interface to compare the different axes. Advice for distinguishing the strongest correlation was given for both the visualization and the sonification. The advice for visual analysis was to look for the “uniformity of the patterns.” The advice for auditory analysis was to listen to “how sorted the sounds are regarding their pitch.” At this stage, the participant was also able to confirm that the sound volume was set at an appropriate level based on the tutorial video, which had the same sound volume as in the training part and the test. The tutorial video can be viewed in *Video 3*^3^ which shows the experiment interface for *Sonification* and *Combination*. At the end of the tutorial, the participant was informed that all user inputs would be recorded for the training and test session and that this data would be stored, analyzed, and reported anonymously. No audio or video recordings of the participants were done, and the participant was able to leave the evaluation session at any point.

A training session followed, familiarizing the participant with the experiment interface by providing the same tasks as in the test, while also giving feedback if an answer was correct or incorrect. If the answer was incorrect, the participant would get further attempts at the task until they were correct. 12 training tasks were presented, with four training tasks for each display type, starting with *Visualization*, followed by *Sonification*, and finally using both in *Combination*. The test session started after the training, containing 30 tasks for each display type, with a total of 90 tasks for the three display types. The presentation order of the display types was structured with a Latin square design, such that the participants would be presented with three different orders. The three possible orders were used equally with the participants to even out any learning or order effects. The tasks alternated between negative and positive correlation for every task, with 15 tasks for each type of correlation. When the test was completed, a post-test questionnaire was filled in by the participant which included questions about the experience of using the different display types. An open-ended interview concluded the evaluation to complement the ratings made in the questionnaire, where the participant was asked to share details of their experience after performing the test. We report on the most frequent comments in Section 6.2.

### Apparatus and architecture

The experiment was performed in a closed-off room on a standard desktop computer setup. The setup consisted of a 25″ computer screen with a resolution of 2560 × 1440 pixels, placed at arm’s length from the participant’s seating position. A pair of Beyerdynamic DT 770 PRO headphones was used for sound playback. A mouse was used to navigate the website displaying the experiment interface. The website was presented in full-screen mode to avoid any visual distractions. The experiment was performed in three separate locations but with the same setup.

To connect the sonification with the visualization we used web technologies with a client-server architecture. The server, implemented with Node.js, performs the client communication by exchanging JSON messages via the WebSocket protocol and communicates with SuperCollider by sending messages using the Open Sound Control [90] protocol. On the client side, the experiment platform was built in TypeScript using the Angular framework.

### Participants

**Fig. 5:**
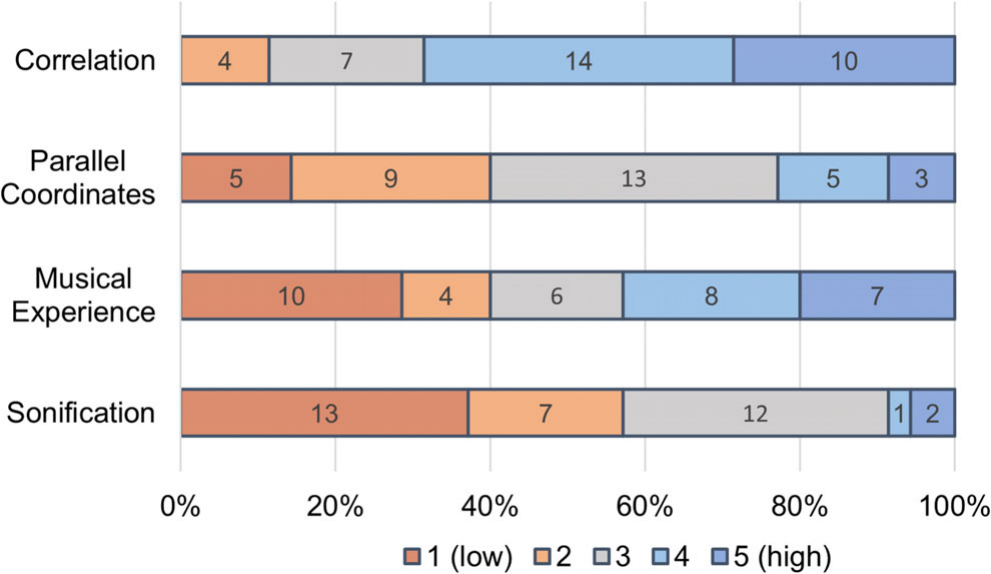
Self-rated knowledge of the experiment participants through a 5-point Likert scale, where 1 corresponded to a low level of knowledge in the subject, and 5 corresponded to a high level of knowledge. See the specific description of the scale items in the evaluation questionnaire as part of the supplemental material

35 participants took part in the controlled experiments (13 female, 19 male, 2 non-binary, 1 gender apathetic) which ranged from 20 to 60 years old (average age of 32.26, *SD* = 9.71). The self-rated knowledge of the participants regarding the concept of correlation, their familiarity with parallel coordinates, their musical experience, and their familiarity with sonification can be seen in Fig. 5. Overall, the participants had more knowledge in correlation and parallel coordinates compared to their knowledge in sonification and musical experience.

## Results from the user evaluation

Statistical analysis was performed on the observed sensitivity thresholds and on the participants’ task completion times. According to two different normality-tests [18, 19, 77], neither sensitivity threshold nor task completion time consistently follow normal distributions. For further analysis, we used a Friedman test [32] and Wilcoxon signed rank tests [89]. Holm-Bonferroni correction [43] for multiple comparisons was used where necessary. In addition to the significance analysis, the effect size measure “Cliff’s Delta” ($\Delta$) [16] is reported.^4^

The *p*-values and $\Delta$-values of the analysis are displayed in Table 1. They provide the results for both main effects (the type of correlation and the type of display) and all potentially interesting pairwise comparisons of conditions. For each participant, both the sensitivity threshold and the task completion time metrics were calculated by averaging the last eight of fifteen responses, i.e., the second half of each staircase. Even though some participants might not have converged towards their individual sensitivity threshold within the first half of each staircase, we confirmed that all results for the sensitivity thresholds reported in Table 1 are robust. The *p*-values and $\Delta$-values would not change in a relevant way if one of the three different metrics was applied: (1) only the last value of each staircase, (2) the average over the last 4 responses, and (3) the average over all 15 responses.

Thematic analysis [10] was performed on the notes from the interviews with the participants by coding the notes based on a number of topics. These topics included comments on their approach for making a decision during the test, how they perceived the sonification, and for which condition they were most or least confident. The comments in each category were then counted by how many times a specific opinion was mentioned, where the most frequent comments are presented as results in Section 6.2.

### Quantitative results

This section presents the quantitative results for the participants’ sensitivity thresholds when they distinguished different strengths of correlations, results with respect to task completion times, and reflections on the influence of prior experience on the results. Kruskal-Wallis tests [50] did not reveal any significant differences regarding the order of presentation of display types (sensitivity: $p > 0.16$; task completion time: $p > 0.08$).

**Table 1:** *p*-values and Cliff’s Delta values for all comparisons regarding the sensitivity thresholds of participants

	Comparisons		*p*	$\Delta$
Main effect 1	Neg	Pos	$\ll$0.01	−0.3
Main effect 2	Vis	Son	$\ll$0.01	−0.69
	Son	Combi	$\ll$0.01	0.63
	Vis	Combi	0.07	−0.11
Pairwise	Vis Neg	Vis Pos	< 0.01	−0.45
	Son Neg	Son Pos	0.28	−0.09
	Combi Neg	Combi Pos	< 0.01	−0.40
	Vis Neg	Son Neg	$\ll$0.01	−0.83
	Vis Neg	Combi Neg	0.15	−0.19
	Son Neg	Combi Neg	$\ll$0.01	0.78
	Vis Pos	Son Pos	< 0.01	−0.57
	Vis Pos	Combi Pos	0.24	−0.10
	Son Pos	Combi Pos	< 0.01	0.48

The metric used for calculation is the average of the last 8 out of 15 responses in each staircase. “$\ll$” means smaller than $10^{-5}$ and “<” is smaller than $10^{-3}$

#### Sensitivity threshold

We found significant main effects of the correlation type ($p \ll 0.01$) and the display type ($p \ll 0.01$) on the participants’ sensitivity thresholds and no significant interaction between the two factors ($p = 0.59$). Pairwise comparisons in Table 1 show differences between correlation types within each display type, and between display types within each correlation type.

##### Main effect 1: correlation type

To study the main effect of the correlation type, we accumulated the data for the display type. The correlation type influenced the participants’ sensitivity threshold significantly (Main Effect 1: $p \ll 0.01, \Delta = -0.3$). Being presented with negative correlations, the participants reached a lower threshold (i.e., they had better sensitivity) than with positive correlations displayed to them.

##### Main effect 2: display type

To study the main effect of the display type, we accumulated the data for the correlation type and ran a Friedman test ($p \ll 0.01$), followed by Wilcoxon signed rank tests for the three pairwise comparisons. While there is no significant difference between *Visualization* and *Combination* (*p* = 0.07, $\Delta = -0.11$), both of them significantly differ from *Sonification* (Vis: $p \ll 0.01$, $\Delta = -0.69$; Son: $p \ll 0.01$, $\Delta = 0.63$).

##### Interaction

**Fig. 6:**
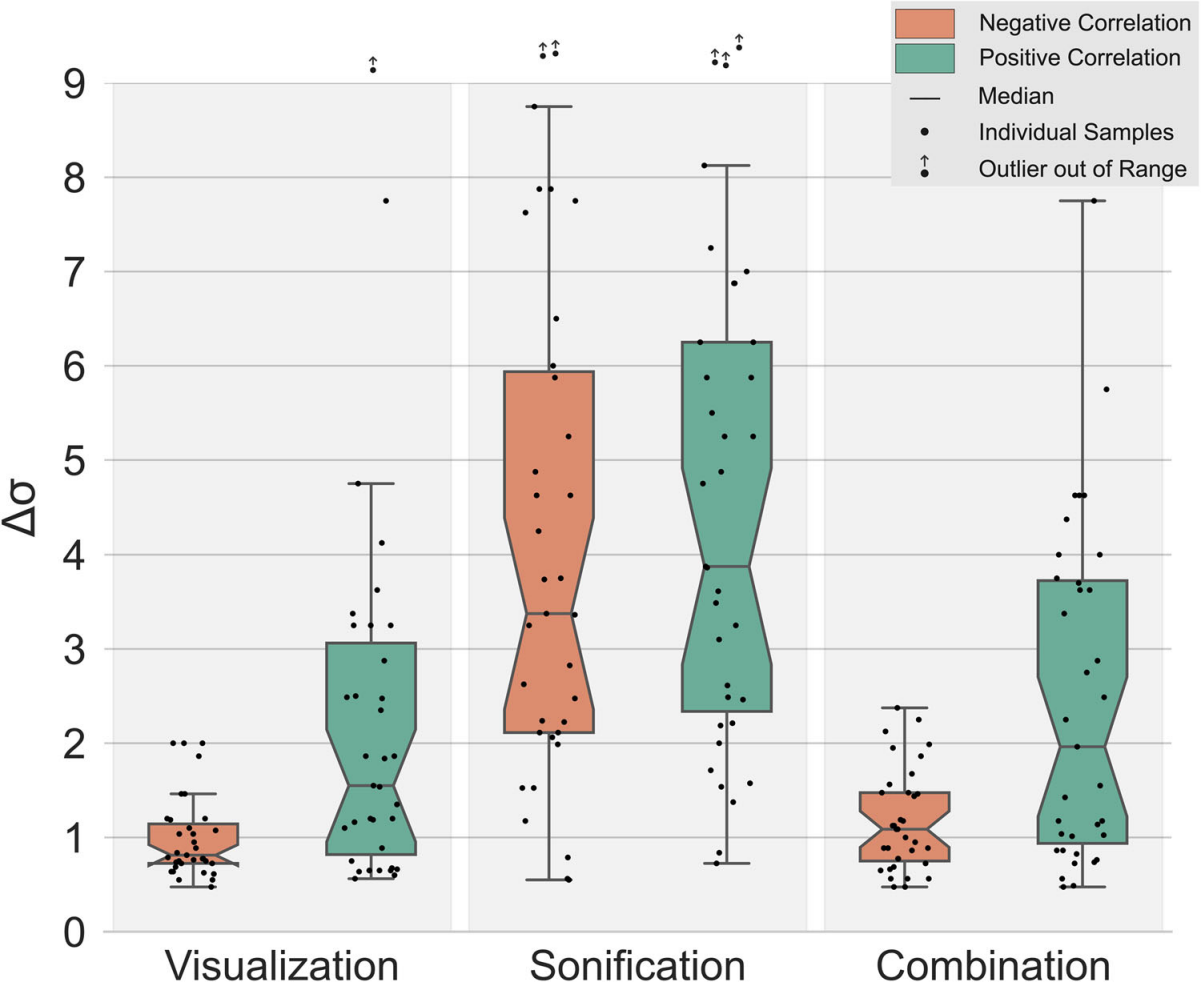
The sensitivity thresholds for 35 participants and six different conditions are displayed as box plots. Participants are generally more sensitive using *Visualization* and *Combination*. Only for *Sonification*, the sensitivity threshold is not affected by the type of correlation

Using an aligned rank transform [40] enabled the application of a repeated measures two-way ANOVA, which did not reveal any interaction between the independent variables “Correlation type” and the “Display type” (*p* = 0.59) [5, 28]. In line with this result, the distributions show parallel trends for positive and negative correlations, i.e., the display type affects both correlation types similarly (see Fig. 6).

##### Pairwise comparisons

Within the two display types *Visualization* and *Combination* a Wilcoxon signed rank pairwise comparison revealed a significant difference between negative and positive correlations (Vis: $p < 0.01,~\Delta = -0.45$; Combi: $p < 0.01, ~\Delta = -0.40$). Using *Visualization* and *Combination* participants were able to distinguish significantly smaller differences whenever they were presented with negative correlations. For *Sonification*, a pairwise comparison revealed no significant difference between negative and positive correlations ($p=0.28, \Delta = -0.09$).

Within the group of negative correlations, pairwise comparisons revealed significant differences between *Visualization* and *Sonification* ($p \ll 0.01, \Delta = -0.83$), between *Sonification* and *Combination* ($p \ll 0.01, \Delta = 0.78$), but not between *Visualization* and *Combination* ($p = 0.15, \Delta = -0.19$).

Within the group of positive correlations, pairwise comparisons revealed significant differences between *Visualization* and *Sonification* ($p < 0.01, \Delta = -0.57$), between *Sonification* and *Combination* ($p < 0.01, \Delta = -0.48$), but not between *Visualization* and *Combination* ($p = 0.24, \Delta = -0.10$).

#### Task completion time

Regarding the participants’ task completion times, we found a significant main effect only of the correlation type ($p \ll 0.01, \Delta = -0.18$). The difference is small and is dominated by the results of *Visualization* and the *Combination* conditions. None of the other comparisons are robust against changing the metric from the average of the last 8 values. In most cases other metrics would lead to not significant differences and small effect sizes, hence, we do not consider them reportable. Table 2 shows the medians and standard deviations of the participants’ task completion times, all being in similar ranges.

#### Self-rated knowledge analysis

In the questionnaire accompanying the experiment, participants rated their prior experience regarding four topics: the concept of correlation, parallel coordinates plots, their musicality, and the method of sonification (see Fig. 5).

Whether prior knowledge was affecting the sensitivity threshold of the participants was studied by comparing groups of participants with low experience to groups with high experience. The grouping was done such that the data would be distributed as balanced as possible between the two groups.^5^ A visual comparison of box plots and the analysis of Cliff’s Delta values revealed that the only condition potentially affected was *Sonification* for negative correlations ($\Delta = -0.35$ for sonification experience, $-$0.3 for musical experience, $-$0.14 for experience with parallel coordinates, and $-$0.36 for correlation experience). All other effect sizes were too small to be considered relevant. As the sample size of the two compared distributions is small the reliability of these results is vague. Therefore, the phenomenon we see in the data can only be considered an indication of a possible effect.

**Table 2:** Median values for sensitivity thresholds and task completion times ± their standard deviations for the six different conditions

Condition		Sensitivity thresholds [$\Delta \sigma$]	TC-times [s]
Visualization	Neg	$0.8 \pm 0.4$	$14.6 \pm 5.9$
	Pos	$1.5 \pm 2$	$18.5 \pm 10.3$
Sonification	Neg	$3.4 \pm 3.4$	$14.8 \pm 4.9$
	Pos	$3.9 \pm 3.8$	$17 \pm 5$
Combination	Neg	$1.1 \pm 0.5$	$16.3 \pm 4.8$
	Pos	$2 \pm 1.8$	$18.7 \pm 8.9$

The metric used for calculation is the average of the last 8 out of 15 responses in each staircase

In addition to the grouping of sensitivities, a global cluster analysis of all participants revealed that four participants showed especially different behavior. While these four participants answered very differently than the more homogeneous rest of the participants, they also rated their sonification experience and musical experience as low. For the sake of transparency and a diverse sample of participants, we decided to not exclude those four participants from any statistical analysis presented in this study. Nevertheless, we did run all the analyses also with a sample of only 31 participants, in general revealing the same phenomena as the sample with 35 participants: The outliers for the *Sonification* conditions in Fig. 6 would disappear, i.e., it is the same distinct group of people causing the especially low sensitivity threshold observations when it comes to sonification. With the four participants excluded, the comparison of the display types *Sonification* and *Combination* would be significantly different ($p = 0.04$) but still show a small effect ($\Delta = -0.14$).

### Subjective ratings and experiences

**Fig. 7:**
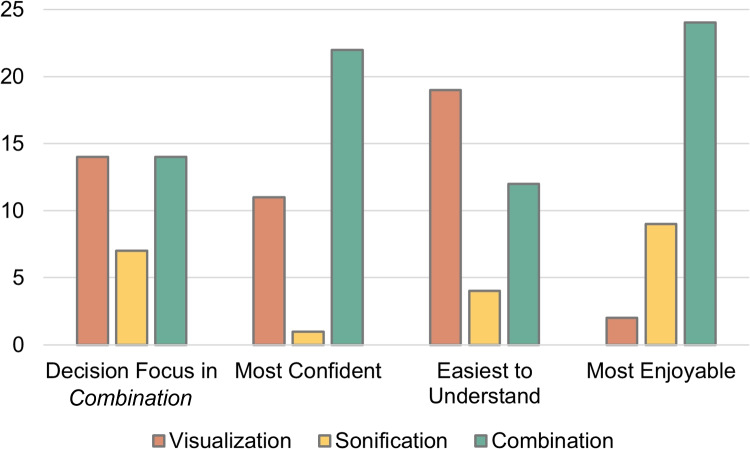
Number of responses to the post-test questionnaire regarding the preference of display type. When using *Combination*, 14 participants decided to focus more on the visualization, 6 focused more on the sonification, and 14 used both for their decision

The results from the post-test questionnaire can be seen in Fig. 7, where the participants answered which modality was preferred for making a decision when using *Combination*, which display type the participant was most confident using, which display type was easier to understand, and which display type was the most enjoyable to use. Overall, the participants felt most confident when using *Combination* (22 of 35), but reported that *Visualization* was easier to understand (19 of 35), and that *Combination* was the most enjoyable display type to use (24 of 35). The participants rated that they either used just the visualization or a combination of the visualization and sonification to reach a decision for their answer (both 14 of 35).

The open-ended interviews provided additional insights into the participants’ experience. When comparing the difficulty of analyzing the two types of correlations across the display types, eleven participants explicitly stated that using *Visualization* for negative correlations was the easiest to interpret. For *Sonification*, four participants stated that the positive correlation and negative correlation were equally difficult to interpret, while three other participants stated that the positive correlation was easier to interpret compared to the negative correlation for *Sonification*. Three participants stated that the negative correlations were easier to interpret across all of the three display types.

Regarding the decision strategy when using *Combination*, eight participants stated that they used the visualization for the first and easier tasks, and when tasks became more difficult they started to also use the sonification in their decision-making. Seven participants stated that they used the visualization to make an initial decision, and then used the sonification do double-check their decision. One participant mentioned that *“the audio helped with intuitive decisions and the visuals helped with analytic decisions.”*

Regarding the sonification, five participants expressed that they were not confident with how they were supposed to interpret the sonification. A participant with high self-rated musical experience mentioned that they thought more about the harmony of the sound while analyzing with the sonification, referring to the tonal relationship between the first and last auditory marks. Some participants mentioned how the sonification affected them emotionally, where one participant stated that *“it felt satisfying to hear the strongest correlation,”* and *“I found Visualization way more tedious/boring – with the sound the time of the task felt shorter.”*

### Summary of the results

The analysis presented in Section 6.1 shows that participants were able to identify the strongest correlation with all three types of display but with different sensitivity thresholds. For *Visualization* and *Combination*, the participants reached a lower sensitivity threshold, i.e., they had better sensitivity (see Fig. 6 and Table 2). With negative correlations, participants were even able to distinguish datasets that only differed by an amount of $0.8\pm 0.4\%$ added noise and below. Using *Sonification* enabled participants to reach a threshold of 3–4% of added noise (with standard deviations of also 3–4%). This is in line with the subjective feedback from several of the participants stating that using *Visualization* for negative correlations was the easiest to interpret, mostly because they were able to compare the density of lines in the central area between the two axes. As the analysis did not show a statistically significant difference between the two display types *Visualization* and *Combination*, it can be assumed that, when having both modalities available, the visualization was the dominant representation to take a decision. While the thresholds for *Visualization* and *Combination* were dependent on the type of correlation, the thresholds for *Sonification* did not show a dependency on the type of correlation. A general user, comparing non-adjacent axes using sonification, would be similarly sensitive to changes of both positive and negative correlation coefficients.

The use of the three display types did not influence the task completion time significantly. While previous research showed higher task completion times with sonification [71], such a phenomenon did not appear in this study. The type of correlation, on the other hand, was influential on the participants’ task completion times. Negative correlations did lead to slightly but significantly faster responses than positive correlation examples, which is a plausible outcome with negative correlations resulting also in better sensitivity thresholds.

In the beginning of the user evaluation, the participants were asked to report on their prior experience using sonification and on their musicality. Our results indicate that the participants’ prior knowledge of these two was influential on only one specific condition: using *Sonification* to distinguish negative correlations. Participants with more sound experience were more sensitive than the ones without prior knowledge ($\Delta = -0.35$ for sonification experience and $-0.3$ for musical experience). It is plausible that prior experience and familiarity with sound affect the sensitivity threshold of participants with the sonification conditions, but while the phenomenon seems to exist for negative correlations, it does not for positive correlations. Masking effects as they are described by Schnupp et al. [75] might be relevant for the explanation of such a phenomenon. As the sound design, however, is based on semitones, it is not reasonable that masking effects are solely responsible for the phenomenon that we see in the data, and future research is needed to explain this observation.

The subjective ratings from Section 6.2 show that using *Combination* made participants more confident in their answers, and it was also the most enjoyable display type to use. The answers from the interviews indicate that participants used the sonification either to form an intuitive decision or to double-check their decision based on the visualization. Furthermore, the interviews indicated that sonification has the potential not only to serve as an analysis method but also to increase the engagement and emotional involvement.

## Discussion

The evaluation of the present design was conducted by combining three approaches described by Isenberg et al. [45]. First, we studied the user performance (UP) by collecting data on the sensitivity, task completion times, as well as number of interactions done by subjects to find an answers. Second, we studied the user experience (UE) by collecting data on the subjects’ confidence and enjoyment while using Parallel Chords. Third, we provide a qualitative result inspection (QRI) by describing the prototypical patterns and presenting a usage scenario.

To the best of our knowledge, we are not aware of a study that showed the dependency of users’ sensitivity for differences in correlations on the direction of the correlations using parallel coordinates plots. The perceptive advantage of negative correlation patterns cannot be considered a surprise to the visualization community. Nevertheless, our data confirms that phenomenon. While we see a dependency on the type of correlation in the visualization condition, we could not observe such a dependency in the sonification condition. Again, we are not aware of a study in the sonification literature that tested differences in sensitivity for very similar correlation strengths. It is necessary to put this result into context: For the chosen sound design (which is in general a widely used one) and for the chosen base level of correlation around $$r = 0.95 $$ ($$\sigma = 0.07$$), we are able to report a sensitivity threshold between three and four percent of added Gaussian noise. Furthermore, we were able to show that the type of correlation (positive or negative) does not affect the sensitivity when participants use the sonification. To understand these phenomena in more detail, a follow-up study would be necessary, testing sensitivities at different base correlation levels.

When relating our evaluation results to existing literature in a more general sense, we can see that some align with our results, while others deviate. Similar to our results, Stahl and Vogt  [78] showed, in contrary to their initial hypothesis, that the audiovisual condition of an experiment with serial spatial stimuli did not show improved learnability of spatial positions compared to a visual-only condition. Similarly, when augmenting a visualization for a guidance task with sonification, Roodaki et al. [73] conducted evaluations which showed that participants performed better when using the visual-only technique in comparison with the audiovisual technique. On the other hand, a study of Rönnberg and Johansson [72] showed that participants gave more precise answers but that the response time was longer when using an audio-visual display, compared to a visual-only display. In our study, we observed lower sensitivity and no reportable difference in response time. The results of the experiment by Flowers et al. [31] also suggest that there is a cross-modal equivalence of visual and auditory scatterplots for exploring bivariate data samples, which strengthens the motivation of presenting a scatter plot in the auditory domain.

Regarding the subjective aspect of the evaluation results, most participants rated that the visualization was the easiest to understand out of the three display types. This can be associated with the challenge for sonification as a data representation technique being less known and used in everyday life [6]. The lower sonification literacy can affect the amount of training needed for participants to get accustomed to the mappings [74]. This might also have influenced the participants’ strategy when taking a decision in the experiment, as several of the participants stated that they focused on the visualization as their primary method for reaching a decision, while the sonification was used to double-check or only used for the more difficult tasks. Still, the sonification influenced the overall experience of performing the experiment tasks, considering that most participants felt more confident and found the tasks more enjoyable when using the visualization together with the sonification compared to using the visualization by itself. These findings are emphasized by some of the participants’ feedback of the sonification making them more involved and satisfied when hearing the strongest correlation, which, in turn, suggests that the sonification positively contributes to the user experience. Assessing enjoyability acts as an initial exploration of whether the use of *Parallel Chords* can promote more engagement of the user, which could extend the use of the tool beyond analytics and towards public engagement as well for future applications.

### Applicability

The results from the present study show that it is possible to distinguish between different strengths of correlation using all three display types but with different sensitivity thresholds. Whenever the participants were able to use the visualization they reached lower sensitivity thresholds, i.e., they performed better. The participants’ ability to correctly interpret the presented datasets with sonification shows the potential for applicability for other purposes since distinguishing between the strength of correlations is a more complex task than just being aware of the existence or the quality of a pattern. Therefore, the study results suggest *Parallel Chords* will be suitable at conveying complementary and more high-level information about a dataset. Rather than distinguishing between the strength of correlations, *Parallel Chords* can be used to convey an overview of a dataset, to make the user aware of the existence of patterns in the dataset. As demonstrated with prototypical patterns in Section 4, it is possible to distinguish between several types of patterns while using the same sonification mappings. This can be beneficially used when searching for patterns occurring between non-adjacent axes in a parallel coordinates plots, as demonstrated in the usage scenario in Section 4.1, which can alleviate the challenge of axis ordering with parallel coordinates.

As an audio-analytics design, *Parallel Chords* can draw from the advantages of both sonification and visualization. The sonification can extend a traditional parallel coordinates plot by giving non-adjacent axes information while keeping the same view in the visualization, which could be beneficial when considering that interactive reordering costs time and cognitive resources [51]. On the other end, other multi-dimensional visualization conveys more dimensions by displaying more plots of the data, such as the scatter plot matrix. With our design, it is possible to display additional dimensions while still keeping the same screen estate, which can be more suitable for smaller visual interfaces. By conveying the data through sound, it offers a temporal aspect which can lead to new insights of the data. In the context of our design, the temporal perspective of sonification could therefore aid domain experts in the analysis of multi-dimensional time-series data. Moreover, the fact that sound can be perceived all around the user leads to that it can be utilized in settings where the visual modality is limited. Monitoring tasks are one such situation, where multi-dimensional temporal data can be conveyed to the user through the focused visual display as well as in the periphery through the sonification. This is also the case in virtual reality and other immersive environments, where the sonification component of our design could guide the user to interesting attributes in the dataset, which would otherwise be occluded or be out of sight in a 3D environment. The *Parallel Chords* design could also be of use in conventional data analysis and decision-taking processes, as they are common in industrial design contexts. An example for the use of parallel coordinates supporting decision-taking between multi-criteria alternatives is described by Cibulski et al. [14].

When relating our design to other similar designs in the literature, we find examples of how our design provides a different approach. The sonification design of Rönnberg and Johansson [72] extends a parallel coordinates display with sonification to support a user in identifying areas of different densities. In comparison to their design, ours sonifies the data items individually. While the density display provides information about one specific phenomenon, *Parallel Chords* is designed to help users identify several different types of patterns in their data. Parson et al. [62] used a sonification approach that treated a parallel coordinates plot as a waveform, and changed the timbre of the sonification based on the average value of the attributes of the dataset. While this facilities an overview of the dataset, it does not allow the user to focus on specific attributes with the sonification. Through the *Parallel Chords* design it is possible to get specific attribute information by selecting different axes in the parallel coordinates plot.

### Limitations

*Parallel Chords* is not intended to increase the amount of data that can be displayed using parallel coordinates, rather it aims to support the use of parallel coordinates for data exploration. Generally, *Parallel Chords* representing data both spatially and temporally affects the scalability of the design in two ways. While the conventional limitations regarding the visual overplotting need to be considered, *Parallel Chords* also is limited by temporal constraints. We evaluated the design by sonifying 300 items with their onsets happening within one second. Considering three to four seconds as the maximum feasible time for effective sonification, the design scales up to about 1000 data items. Considering different sound designs with, e.g., shorter sounds or additional spatial positioning of auditory marks could increase the scalability of the design.

While one central application of sonification concerns the accessibility of visual display, this was not the focus of this study. Nevertheless, the results regarding sensitivity threshold and the different auditory patterns can inspire the design of accessible visualizations. The design implications for the sonification are not limited to parallel coordinates plots in that regard.

The method to generate the data for the controlled experiment of this study also implies one of its limitations. Some participants were able to distinguish datasets that only differed by a very small amount of added Gaussian noise, i.e., by a standard deviation of only 1% and below. Such small differences can not only be too small to be perceived as different, but also to be considered as statistically different at all. To make sure to not ask participants to detect differences between two statistically equal datasets, we re-generated the staircase datasets whenever their Pearson *r* values would not be monotonically decreasing over the course of the staircase. It is unlikely that users would ever want to distinguish such small differences between correlations ( $\Delta \sigma < 1\%$) using a parallel coordinates plot. Therefore, when participants were able to distinguish such small differences, they can be considered as perfectly sensitive.

To rule out the possibility of systematic higher sensitivity thresholds due to our sonification design, we also studied the potential influence of the chosen MIDI quantization of the *pitch axis* on the evaluation results. A comparison between the Pearson r correlation coefficients with and without the 36-step quantization of one of the axes showed a neglectable difference. We can conclude that the observed sensitivity thresholds with sonification, and therefore also the evaluation results, were not significantly impacted by the MIDI quantization.

## Conclusion

We presented *Parallel Chords*, an audio-visual analytics design for parallel coordinates that combines visual and auditory displays to aid the user in finding and determining patterns in multivariate data. Through a set of prototypical patterns, we demonstrated how the sonification of *Parallel Chords* can be interpreted to identify patterns together with a parallel coordinates plot. With a usage scenario of a real dataset, we showed how *Parallel Chords* can be used to convey patterns between non-adjacent axes. The results of a controlled user evaluation showed that participants were able to distinguish differences of correlations, but with different sensitivities when only using visualization or sonification, and when using a combination of both.

While in this article we focused on only one of many possible designs to combine parallel coordinates plots and sonification, future work will cover several other aspects. In this study, synthetic data was used as stimuli for participants in the user evaluation to act as a first step to validate *Parallel Chords*. Future experiments will need to use datasets and the expertise from data analysts of different domains to evaluate the real-world applicability of the current design. To allow for a more extensive exploratory data analysis approach, *Parallel Chords* can be extended to more efficiently compare several non-adjacent axes. This could be done by sequentially sonifying every pairwise relation of one axis through one interaction of the user. Alternatively, the variables of a dataset could be mapped to individual auditory channels (like pitch, spatial position, timbre, duration, and loudness) to enable a user to become aware of the existence of patterns in their data by only listening to the sequence of polylines once. A controlled experiment would then reveal if a user would also identify a correlation by hearing a complex sound moving from one speaker to the other, or by the sounds getting louder over time.

The results suggest that *Parallel Chords* can be a useful audio-visual analytics tool, even if more research is needed to fully explore and evaluate it. The work has not only led to novel knowledge about audio-visual analytics but also, to some extent, bridged the distance between the visualization and the sonification research communities.

## Supplementary Information

Below is the link to the electronic supplementary material.

Supplementary file 1 (mp4 26216 KB)

Supplementary file 2 (mp4 141562 KB)

## Data Availability

The datasets generated during and/or analyzed during the current study are available in the osf repository, https://osf.io/z9vnw/?view_only=bac1e61f5b1e4b3fb7c08720ef5d7355

## Footnotes

1 Video of prototypical patterns: https://www.youtube.com/watch?v=T2n7JV9Qvog

2 https://www.youtube.com/watch?v=BYZVYHx56P8 - The displayed data is the iris dataset [29]

3 Tutorial video: https://www.youtube.com/watch?v=fp6dOphJ5FI

4 $\Delta$-values around 0.11 can be considered as “small effects,” $\Delta$-values around 0.28 as “medium effects,” and $\Delta$-values around and above 0.43 as “large effects” [84].

5 Grouping for correlation ratings 2|3 vs. 4|5; parallel coordinates 1|2 vs. 3|4|5; musical experience 1|2 vs. 3|4|5; sonification 1|2 vs. 3|4|5.
